# Cerebro-Spinal Meningitis

**Published:** 1872-03

**Authors:** J. G. Westmoreland

**Affiliations:** Atlanta, Ga., Professor of Materia Medica and Therapeutics in Atlanta Medical College


### CEREBRO-SPINAL MENINGITIS.

BY J. G. WESTMORELAND, M.D., ATLANTA, GA.,
Professor of Materia Medica and Therapeutics in Atlanta Medical College.

This universally and justly-dreaded malady probably takes its origin in electrical, thermal or other atmospheric peculiarities, which, affecting whole communities sometimes, is called epidemic. The influence thus leading to local irritation and inflammation in various parts of the respiratory apparatus and nervous centers is called *influenza.* Names are given to the local manifestations in accordance with the part affected. Thus laryngitis, tonsilitis, etc., indicate the part concerned. The *degree* of derangement is denoted also by definite terms, or names given to the disturbance. For example, irritation or slight inflammation of the mucous membrane lining the air-passages is called "catarrh" or "*bad cold.*" When more decided and violent, producing fever and other inflammatory symptoms, bronchitis, laryngitis, etc., are the names given it. Different degrees of irritation also exist in disturbance about the nervous centers from this influence. The results of irritation or moderate inflammation connected with the meninges of centers or nervous theca, are known as "crick in the neck," "stich," "lumbago," "sciatica," "pleurodynia," etc.; while decided violent inflammation, leading to convulsions, paralysis, etc., takes the name meningitis, which indicates the existing pathological condition.

Other parts about the head are often affected by this same influence. The ears, frontal sinus and maxillary antrum are subject to painful and protracted disease from an epidemic of influenza. Each separate epidemic has the tendency to particular parts.

The "*Tyler-grippe*" of 1841 was perhaps the most extensive epidemic in America for the last forty years. This affected the nasal cavities and antrums, and in some instances the ears, throat and bronchia. Some epidemics of the kind are characterized by tonsilitis, others by laryngitis, and occasionally meningeal trouble marks its course.

Meningitis is found to exhibit various degrees of violence, and to this fact is attributable the marked difference in the symptoms as given by practitioners. That case in which the patient is speedily thrown into convulsions and coma is no more certainly meningitis than one which, for a day or two complains of headache, pain and rigidity of the neck and back, before spasm or even delirium is well-established. Those who confine the disease to those violent and malignant cases in which unconsciousness and convulsions are the first symptoms discovered, and which terminate fatally in twelve hours, discourage practical investigation in the treatment of this alarming disease. Such are, in many instances, uncontrollable; while those in which the inflammation is less violent may be relieved, by prompt and decided treatment, with as much certainty as inflammatory disease of other vital organs. As well might we say that a case of pneumonia is not entitled to the name when not sufficiently extensive or of sufficient violence to prostrate the subject beyond the point of reaction. Under such circumstances, the functions of vital organs are arrested before the establishment of true inflammation. The first stage—that of engorgement—has been sufficient to bring about this result, and the stage of excitement does not come on at all. Hence, in meningitis, when the irritation is so general and excessive that, by mechanical pressure or otherwise, the cerebro-spinal functions are so nearly arrested that coma or convulsions supervene, and the patient often dies from this cause without symptoms of inflammatory excitement. If sufficient vital or nervous power exists to allow unloading of the engorged vessels of the meninges by general depletion, relief may be afforded by this means; but, unfortunately, in some cases, it does not, and counter-irritants, anti-irritants and cerebral stimulants afford the only hope of relief. Opium, in such conditions, tends to allay irritation and relieve the consequent congestion, and also to support, temporarily, the nervous vital functions. Counter-irritants, in the form of cups and prompt vesicants over the occiput and spine, serve for the former of these objects by establishing a similar condition in the skin adjacent to the diseased vital part.

Less violent cases, in which, although great nervous disturbance exists, inflammatory febrile reaction is set up, are subject to more extensive treatment. Depletion by general bloodletting should constitute the first step in the application of remedies. The practitioner should be governed in the amount of blood abstracted by the effect produced upon the patient while in a semi-recumbent or sitting posture. Counter-irritation by blisters is indispensable. Large doses of mercury, for its spanæmic effect in reducing the plastic quality of the blood by the destruction of its fibrin, serve a valuable purpose. It may be given in the dose of forty grains, and should be administered promptly.

Notwithstanding the general use of quinine in the treatment of this disease, it is only in the sequela or results that any benefit seems to be derived from it. After the inflammatory condition has subsided, the cerebro-spinal center is sometimes left in a diseased condition, which keeps up an intermittent fever for a number of days. This yields promptly to the ordinary quantity of quinine.

